# Trending Topics on Coumarin and Its Derivatives in 2020

**DOI:** 10.3390/molecules26020501

**Published:** 2021-01-19

**Authors:** Aitor Carneiro, Maria João Matos, Eugenio Uriarte, Lourdes Santana

**Affiliations:** 1 Departamento de Química Orgánica, Facultade de Farmacia, Universidade de Santiago de Compostela, 15782 Santiago de Compostela, Spain; aitor.carneiro@gmail.com (A.C.); mariajoao.correiapinto@usc.es (M.J.M.); eugenio.uriarte@usc.es (E.U.); 2 CIQUP/Department of Chemistry and Biochemistry, Faculty of Sciences, University of Porto, Rua Campo Alegre 687, 4169-007 Porto, Portugal; 3 Instituto de Ciencias Químicas Aplicadas, Universidad Autónoma de Chile, 7500912 Santiago, Chile

**Keywords:** coumarins, biological applications, drug discovery, fluorescent probes

## Abstract

Coumarins are naturally occurring molecules with a versatile range of activities. Their structural and physicochemical characteristics make them a privileged scaffold in medicinal chemistry and chemical biology. Many research articles and reviews compile information on this important family of compounds. In this overview, the most recent research papers and reviews from 2020 are organized and analyzed, and a discussion on these data is included. Multiple electronic databases were scanned, including SciFinder, Mendeley, and PubMed, the latter being the main source of information. Particular attention was paid to the potential of coumarins as an important scaffold in drug design, as well as fluorescent probes for decaging of prodrugs, metal detection, and diagnostic purposes. Herein we do an analysis of the trending topics related to coumarin and its derivatives in the broad field of drug discovery.

## 1. Introduction

Coumarins are molecules that belong to a very special family. Their conjugated double ring system makes them interesting molecules for different fields of research. Coumarins can be found in industry as cosmetics and perfume ingredients, as food additives, and especially in the pharmaceutical industry in the synthesis of a large number of synthetic pharmaceutical products [1]. This last application is the main focus of our overview.

**Figure 1:**
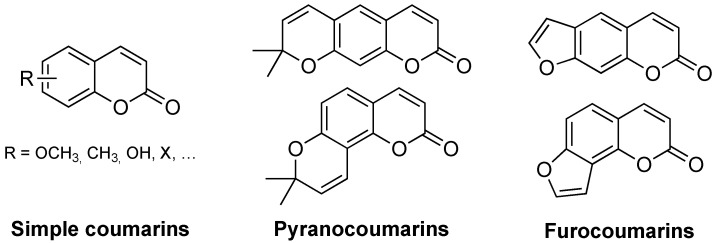
Basic classification of coumarins: chemical structures of the three main classes.

Coumarin (Figure 1) is found in nature in a wide variety of plants, particularly in high concentration in the tonka bean (*Dipteryx odorata*). It can also be found in sweet woodruff (*Galium odoratum*), vanilla grass (*Anthoxanthum odoratum*), and sweet grass (*Hierochloe odorata*), among others. This explains the great interest in the extraction and characterization techniques of natural coumarins, and in the synthesis of their derivatives. In addition, the simplicity of its chemical backbone is very attractive, as well as the reactivity of the benzene and pyrone rings. Conjugated double bonds are responsible for an electronic environment that plays a very important role in this family of compounds.

This review is based on the most relevant literature that comprises new data from recent research articles and overviews on the development of new therapeutic solutions and fluorescent probes based on the coumarin scaffold. The research articles and reviews organized to prepare this manuscript have been compiled from various electronic databases, including SciFinder, Mendeley, and PubMed. The latter was the main source of information, due to its specificity in the biomedical field.

## 2. Discussion

Searching for the word “coumarin” in Mendeley, PubMed, and SciFinder in early November 2020, and filtering by year “2020”, more than a thousand references appeared. Another search was conducted in late December to include as much information as possible in this manuscript. A diversity of journals from different fields publishes research articles and reviews related to the biological interest of both plant extracts containing coumarins and/or synthetic molecules based on this scaffold. Hybrid molecules containing different pharmacophores [2,3] like piperazines or pyrazolines [4] are at the top of the list. For simplicity, in the current review the information is organized taking into account the potential pharmacological/biological applications of coumarin derivatives.

### 2.1. Anticancer Activity

**Figure 2:**
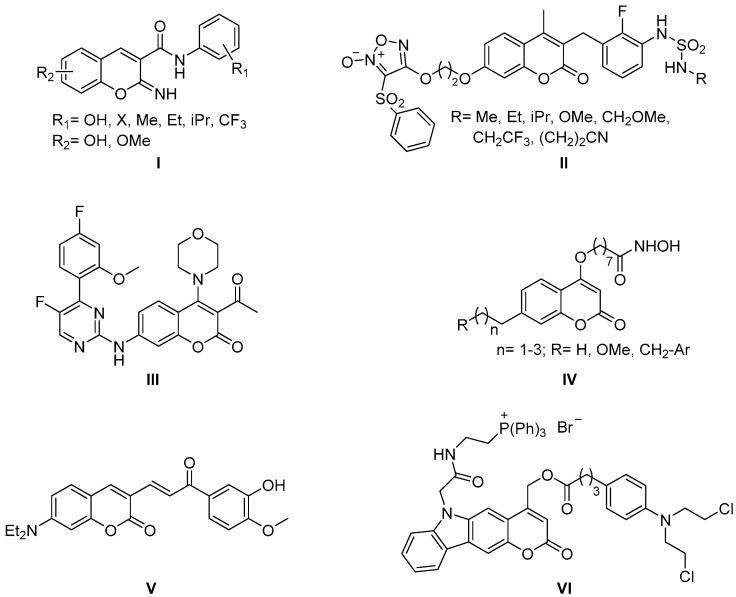
Structures of coumarin derivatives as anticancer agents.

The activity of coumarins as anticancer agents is at the top of reviews published in 2020 [5,6,7,8], as well as research papers. Potent inhibitors (Figure 2, general structure **I**) of aldo–keto reductase (AKR) presenting an iminocoumarin scaffold, with activities between 25 and 56 nM, have been described for the treatment of prostatic cancer [9]. The design of sulfamide 3-benzylcoumarin hybrids bearing an oxadiazole ring at position 7 (Figure 2, general structure **II**) has allowed the preparation of new multitarget mitogen-activated protein kinase (MEK) inhibitors and nitric oxide (NO) donors, both with antiproliferative properties [10]. In other cases, the anticancer profile has been directed to other targets. Such is the case of new inhibitors of cyclin-dependent kinases, specifically CDK9, designing hybrids that incorporate an aminopyrimidine fragment to coumarin, both pharmacophores of known activity on these therapeutic targets [11]. We highlight here compound **III** (Figure 2), with high activity and selectivity for these receptors in comparison with other kinases.

Other important targets for cancer treatment, especially lymphomas, are histone deacetylases (HDACs). A series of coumarins (Figure 2, general structure **IV**) exhibiting a hydroxamate structure similar to HDACi vorinostat (SAHA) has been published [12]. The compounds show inhibitory activity in the nanomolar range, being higher in the case of propyl or methoxypropyl derivatives.

In addition, it is worth highlighting the design of hybrids in which one part of the molecule provides fluorescent properties, and another provides therapeutic action (theranostic). Such is the case of the fusion of a 7-aminocoumarin fluorescent ring with a chalcone fragment (Figure 2, compound **V**). This molecule is an inhibitor of thioredoxin reductases (TrxRs), presenting high antitumor activity (IC_50_ = 3.6 μM), and is also used as a diagnostic agent [13]. Coumarin scaffold fluorescence is being explored extensively in biomedicine, as described at the end of this review.

The preparation of photo-triggered drug delivery systems (PTDDSs, Figure 2, general structure **VI**) has also been described, in which the chlorambucil pharmacophore is incorporated into more complex carbazole–coumarins (electron donor and electron acceptor fragments, respectively), carriers of a mitochondrial triphenylphosphonium ligand. This system allows, by irradiation, the controlled release of the chemotherapeutic agent [14].

Finally, coumarins are widely used as ligands in the formation of metal complexes, as described in a very recent review [15] focusing on their application as anticancer agents. Such is the case of complexes with platinum, palladium, gold, copper, or ruthenium, many of which are also used as described below, in the design of antimicrobial agents.

Within the group of compounds with anticancer activity, coumarins exhibiting an antiglioma profile may be highlighted. Simple coumarins such as osthole, umbelliferone, esculin, and 4-hydroxycoumarin, combined with sorafenib (a kinase inhibitor drug approved for the treatment of primary kidney cancer, advanced primary liver cancer, FLT3-ITD positive acute myeloid leukemia (AML), and radioactive iodine-resistant advanced thyroid carcinoma) were studied [16]. The same group also studied a combination of the same simple coumarins with temozolomide (used in the treatment of brain tumors such as glioblastoma multiforme or anaplastic astrocytoma) [17].

### 2.2. Antimicrobial Activity

**Figure 3:**
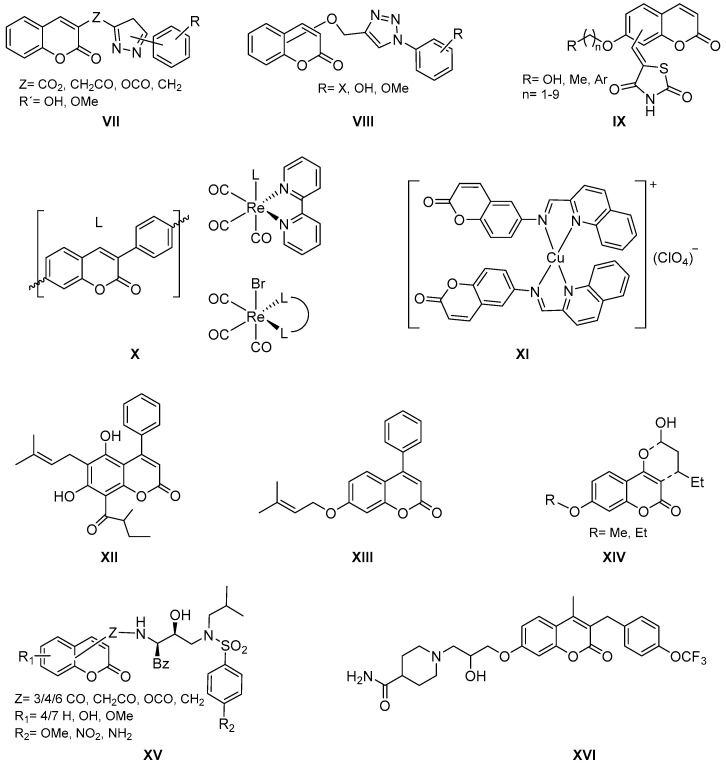
Structures of coumarin derivatives as antimicrobial agents.

There is also an abundant bibliography related to the interest of coumarins as antimicrobials. Most of the projects are still inspired by the classic antibiotic novobiocin. There are several works in which antibacterial activity is found due to the presence of an azole ring introduced in different positions of the coumarin system. Articles have been published recently on the antibacterial activity of azole–coumarins, as well as 3/4/7 substituted arylcoumarins (Figure 3, general structures **VII** and **VIII**), especially active on Gram-positive and negative bacteria according to substitution patterns [15,18,19,20]. In other cases, thiazolidinedione–coumarin hybrids have been described (Figure 3, general structure **IX**) that show activity on methicillin-resistant *Staphylococcus aureus* (MRSA) [21].

Interestingly, coumarin metal complexes also show antibacterial activity. Such is the case of 3-arylcoumarins that present general structures **X** (Figure 3), coordinated with Re(**I**), active against MRSA in nanomolar concentrations [22]; or the complexes of general structure **XI**, a coordination of coumarin–quinoline hybrids with Cu(**I**), with activity against *Flavobacterium psychrophilum*, a Gram-negative bacterium that causes significant septicemia in fish, causing devastating economic problems in aquaculture [23].

In addition to the antibacterial activity, in a recent and comprehensive review on coumarins, activity against protozoa of the genus *Leishmania* was described [24]. The most promising compounds are prenylated, glycosylated, furan/pyranocoumarins, or simple hydroxy- or methoxy-substituted coumarins, along with the natural coumarin mammea A/BB (Figure 3, structure **XII**). Derivatives of this natural product have been prepared and substitutions at positions 6 and 8, as well as the phenyl ring at position 4, turned out to be mandatory for the studied activity. This structure–activity relationship (SAR) study led to the synthesis of the simplest and most lipophilic analogue **XIII** (Figure 3), the most promising member of the group as an antileishmanial agent. Similar structures, some also derived of the *Mammea* genus, have been evaluated against *Mycobacterium tuberculosis*, an activity that also shows simpler synthetic analogues derived from 4-hydroxycoumarin (Figure 3, general structure **XIV**) [25].

Finally, it is worth mentioning two articles reported this year on the design and preparation of coumarin derivatives with potential antiviral activity. This is the case of the dual hybrid inhibitors inspired by the antiviral activity of calanolide, known as reverse transcriptase (RT) inhibitor. With this in mind, dual inhibitors of HIV-1 RT and protease (PR) have been designed, in which the coumarin fragment responsible for RT activity is linked to the fragment of the antiretroviral darunavir, active against PR of the HIV, through different amide, carbamate, or amine linkers (Figure 3, general structure **XV**) [26]. The second case described the introduction of a piperidine ring through a linker in position 7 of the coumarin scaffold, originating compounds with outstanding activity against certain filoviruses such as Marburg virus (MARV) or Ebolavirus (EBOV). From the SAR studied, it is interesting to highlight the role of substitution in *para* position with a trifluoromethoxy group that originated compound **XVI** (Figure 3) with IC_50_ = 0.5 μM and 1.2 μM against EBOV and MARV, respectively [27].

### 2.3. Antioxidant and Anti-Inflammatory Activities

**Figure 4:**
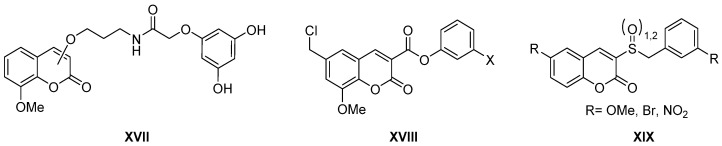
Structures of coumarin derivatives as antioxidant and anti-inflammatory agents.

Although we have found very few publications related to these activities, in some cases the antioxidant activity of coumarin derivatives of both natural [28] and synthetic [29] origin has been reported. This is the case of NOs inhibitors, an activity described for coumarins that bind through different linkers to phenolic fragments capable of acting as radical scavengers (Figure 4, general structure **XVII**), hybrids that can therefore be used in the treatment of immunomodulatory diseases.

Regarding the anti-inflammatory activity, it is worth mentioning a review on the coumarins of natural origin (simple coumarins, prenylcoumarins, furocoumarins, coumestans, and benzocoumarins) with a detailed anti-inflammatory activity due to the activation of the nuclear factor erythroid 2-related factor 2 (Nrf2 factor) that protects cells against stress oxidative [30]. In other cases, the anti-inflammatory activity found for coumarin esters (Figure 4, general structure **XVIII**) as inhibitors of the Kallikrein-related peptidase 9 (KLK9) involved in inflammatory processes of the skin is reported [31]. Finally, the replacement of the carboxylic group by a sulfone or sulfoxide group (Figure 4, general structure **XIX**) gives rise to new inhibitors of cyclooxygenase-2 (COX-2) with activities comparable, in many cases, to indomethacin [32].

### 2.4. Adenosine Ligands

**Figure 5:**
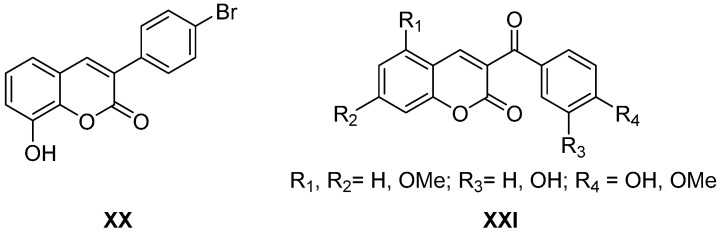
Structures of coumarin derivatives as adenosine ligands.

The affinity of the coumarin system for adenosine receptors has also been published recently. The 3-arylcoumarins (Figure 5, compound **XX**) have been described as antagonists of *h*A_3_ receptors, showing a high affinity (in the low nanomolar range) and selectivity for this subtype [33], while the 3-aroylcoumarins (Figure 5, general structure **XXI**) have been described as dual *h*A_1_/*h*A_3_ antagonists in the low micromolar range [34]. These works are aligned with the already known potential of these derivatives as modulators of the different adenosine receptors, published in the last decade.

### 2.5. Enzymatic Inhibitory Activity: α-Glucosidase, Carbonic Anhydrase, Tyrosinase, Sulfatase, and Xanthine Oxidase

**Figure 6:**
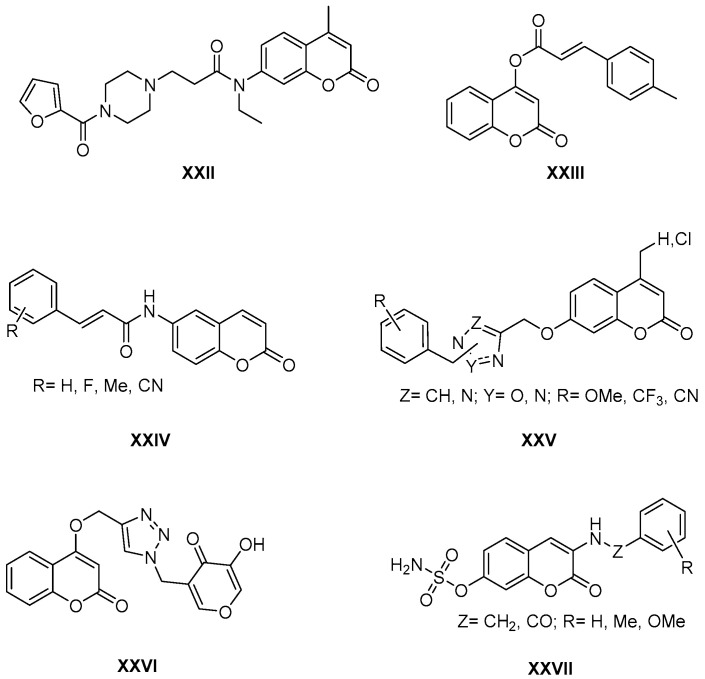
Structures of coumarin derivatives as enzymatic inhibitors.

The activity of coumarin derivatives on α-glucosidase was also reviewed in 2020 [35], in a study in which the influence of the substitution pattern was evaluated, and an important SAR was established. In addition to α-glucosidase, aldehyde dehydrogenase 1A1 (ALDH1A1) is another target for the treatment of diabetes and obesity, and 3-amidocoumarins have been described as inhibitors of this enzyme, with compound **XXII** (Figure 6) being a very promising derivative (IC_50_ = 3.87 mM) [36]. This activity has also recently been found for hybrids of coumarin and cinnamic acid, with compound **XXIII** (Figure 6) being described as a very promising derivative (IC_50_ = 12.98 mM) [37].

Closely related to these structures, works have been published on coumarin derivatives with inhibitory activity on carbonic anhydrase **IX** and **XII**. These are coumarins that incorporate arylacrylamide substituents at position 3 (Figure 6, general structure **XXIV**) that showed inhibitory activity in the nanomolar range [38]. In other cases, anhydrase inhibitory activity was reported for hybrids connected by a methyleneoxy linker at position 7, oxadiazole heterocycles [39] that the same authors extend to the triazole ring (Figure 6, general structure **XXV**) [40].

These last structures are closely related to others that present tyrosinase inhibitory activity in the sub-micromolar range. This is the case of coumarins (umbelliferone and other phenolic analogues) that incorporate a kojic acid fragment through a triazole linker at position 4 (Figure 6, compound **XXVI**), both fragments with demonstrated tyrosinase inhibitory activity [41].

Likewise, the introduction of a sulfamate group in the coumarin scaffold originates a hybrid prototype (Figure 6, general structure **XXVII**) that presents a high inhibitory activity of the steroid sulfatase (best compound of the series with IC_50_ = 0.13 μM), which is of interest in the treatment of hormone-dependent breast cancers [42].

During 2020, 3-phenylcoumarins were also studied as xanthine oxidase inhibitors [43]. Methoxy and nitro substituents were introduced into the framework. The best compound in the series proved to be 3-(4-methoxyphenyl)-6-nitrocoumarin, with an IC_50_ = 8.4 μM, being also non-cytotoxic in B16F10 cells.

### 2.6. Anti-Neurodegenerative Diseases Activity: MAO and AChE/BChE Inhibitors

**Figure 7:**
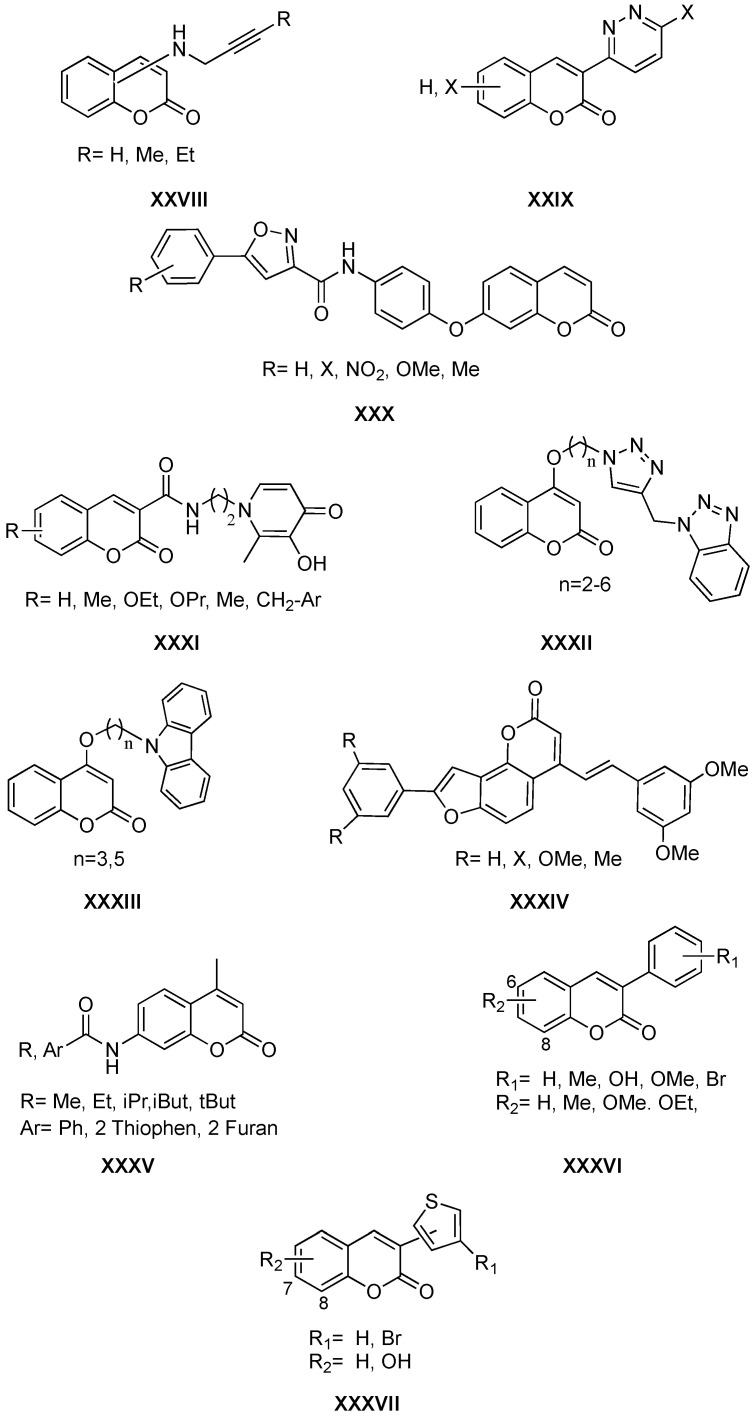
Structures of coumarin derivatives as anti-neurodegenerative diseases agents.

The role played by coumarin derivatives as agents that exhibit biological activities associated with neurodegenerative diseases, such as Alzheimer’s disease, is very important. Throughout this year, a large number of manuscripts related to this field have been found. Due to the multidirectional nature of these diseases, there are also many works on hybrid coumarins directed at different pharmacological targets, such as monoamine oxidase B (MAO-B) or acetylcholinesterase (AChE), amyloid aggregation, or oxidative stress, among others. Hybrids of general structure **XXVIII** (Figure 7) have been described, in which the rasagiline fragment with MAO-B inhibitory activity and neuroprotection properties is incorporated into the coumarin scaffold also with demonstrated MAO-B inhibitory activity, antioxidant, and neuroprotective properties [44]. The incorporation at position 3 of a pyridazine ring (Figure 7, general structure **XXIX**) is another case of hybrid structures as selective MAO-B inhibitors [45]. The incorporation of isoxazole-type heterocycles in carboxamide–coumarins (Figure 7, general structure **XXX**) allowed obtaining derivatives with significant inhibitory activities of AChE/BuChE and beta-secretase 1 (BACE1) [46]. In other cases, taking into account the importance of metals in the pathogenesis of Alzheimer’s disease, a pyridinone fragment (Figure 7, general structure **XXXI**) with iron-chelating properties was incorporated [47].

Other multitarget structures are the benzotriazole–coumarin (Figure 7, general structure **XXXII**) and carbazole–coumarin (Figure 7, general structure **XXXIII**) hybrids [48,49]. In both cases, the molecules show antioxidant activity, as well as AChE and β-amyloid aggregation inhibitory properties. Finally, it is worth mentioning another type of hybrid, this time a furocoumarin that incorporates two fragments of resveratrol (Figure 7, general structure **XXXIV**) [50]. Compounds containing this scaffold present AChE and BACE1 inhibitory properties related to the furocoumarin fragment, and antioxidant (radical scavenging) and COX-2 inhibition, related to the resveratrol [50].

The 7-amidocoumarins (Figure 7, general structure **XXXV**) have recently been published for their potential against monoamine oxidase A (*h*MAO-A), *h*MAO-B, *h*BACE1, *h*AChE, and butyrylcholinesterase (*h*BuChE) [51]. The research project is based on a screening of compounds with potential activity against Alzheimer’s and Parkinson’s diseases, since these multifactorial pathologies share some of their pharmacological targets. Five derivatives of the studied series were described as potent and selective *h*MAO-B inhibitors in the nanomolar range; six turned out to be *h*MAO-A inhibitors in the low micromolar range; one showed inhibitory activity of *h*BACE1, and another one *h*AChE inhibitory activity, both in the micromolar range. In addition to the enzymatic inhibition, all of the studied molecules proved to be non-cytotoxic to neurons in the motor cortex. As a main conclusion, results suggest that by modulating the substitution pattern at position 7 of the scaffold, selective or multitarget molecules can be achieved.

The 3-arylcoumarins are a family of compounds with proven activity on different targets related to neurodegenerative diseases, especially Alzheimer’s and Parkinson’s diseases, the two most prevalent. In the last decade, several manuscripts described very promising activities of this scaffold, both as selective and multitarget compounds. SAR studies were performed, and important conclusions were drawn based on the substitution patterns in the main scaffold. Due to the large number of molecules based on this scaffold currently synthetized and studied as *h*MAO inhibitors, in 2020 a theoretical work was published comparing different QSAR models and docking calculations, in order to predict the *h*MAO-B activity of the 3-arylcoumarins [52]. Based on the predictions, a small series of compounds was synthetized and evaluated against both *h*MAO-A and *h*MAO-B, and the most promising models were validated. Selective activities were found in the low nanomolar range against this isoenzyme for 6 and 8 methyl-substituted 3-arylcoumarins, also presenting methoxy groups or bromine atoms in different positions of the 3-phenyl ring (Figure 7, general structure **XXXVI**). These advancements may represent robust tools in the design of potent and selective derivatives.

Analogues of 3-phenylcoumarins were also published during 2020. The discovery and optimization of 3-thiophenylcoumarins (Figure 7, general structure **XXXVII**) as novel and promising agents against Parkinson’s disease have been described [53]. This study explores, for the first time, the potential of these structures as in vitro and in vivo agents against this disease. The inhibitory activities of *h*MAO-A and *h*MAO-B, antioxidant profile, neurotoxicity in neurons of the motor cortex, and neuroprotection against hydrogen peroxide production were studied. The in vivo effect on locomotor activity was also evaluated by an open field test (OFT) for the most potent, selective and reversible *h*MAO-B inhibitor of the series: 3-(4′-bromothiophen-2′-yl)-7-hydroxycoumarin (IC_50_ = 140 nM). In reserpinized mice pre-treated with levodopa and benserazide, this molecule exhibited a slightly better in vivo profile than selegiline, currently a therapeutic option for Parkinson’s disease. The results suggested that the 7-position substitution of the coumarin scaffold is interesting for enzyme inhibition. Furthermore, the presence of a catechol at positions 7 and 8 exponentially increases the antioxidant potential and the neuroprotective properties.

The neuroprotective effects of xanthotoxin and umbelliferone on streptozotocin (STZ)-induced cognitive dysfunction in rats were evaluated [54]. Alzheimer’s disease was induced in these animals and both compounds were administrated, proving to prevent cognitive deficits in the Morris water maze and object recognition tests. In addition, both compounds reduced the activity of hippocampal AChE and the level of malondialdehyde, increasing the glutathione content. These coumarins also modulated neuronal cell death by reducing the level of proinflammatory cytokines, inhibiting the overexpression of inflammatory markers (nuclear factor κB and cyclooxygenase **II**), and upregulating the expression of NF-κB inhibitor (IκB-α). An attenuation of cognitive dysfunction by these compounds was observed. This effect can at least be attributed to the inhibition of AChE and the reduction of oxidative stress, neuroinflammation, and neuronal loss, opening a new door for these classic coumarins.

### 2.7. Anticoagulant Activity

The classic anticoagulant effect of specific coumarin derivatives, based on acenocoumarol and warfarin, also remains one of the classic applications for this family. During 2020, a review on this topic was published [55].

### 2.8. Fluorescent Probes

In addition to the interest of coumarins as a versatile scaffold in drug design, the important role that this scaffold plays as fluorescent probes to detect metals, enzymes, and biomaterials, among others, should be highlighted [56,57,58,59]. These fluorescent probes have a great imaging potential for the diagnosis of several pathologies.

**Figure 8:**
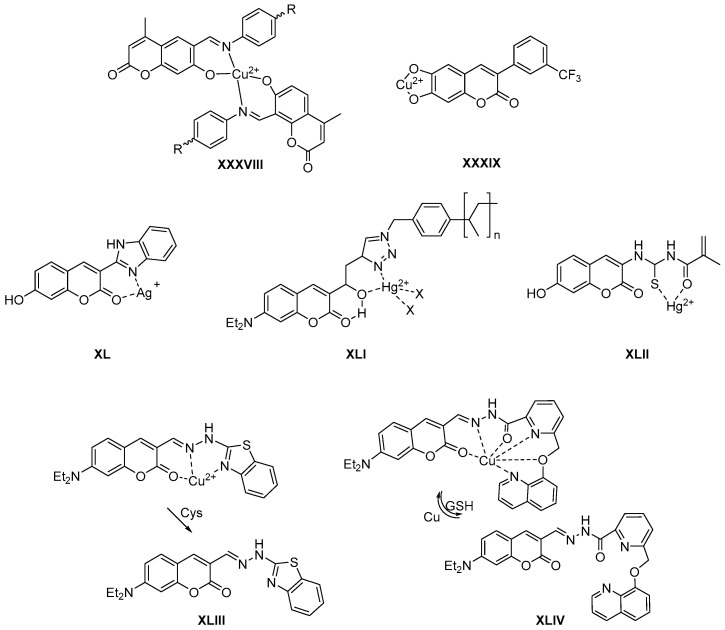

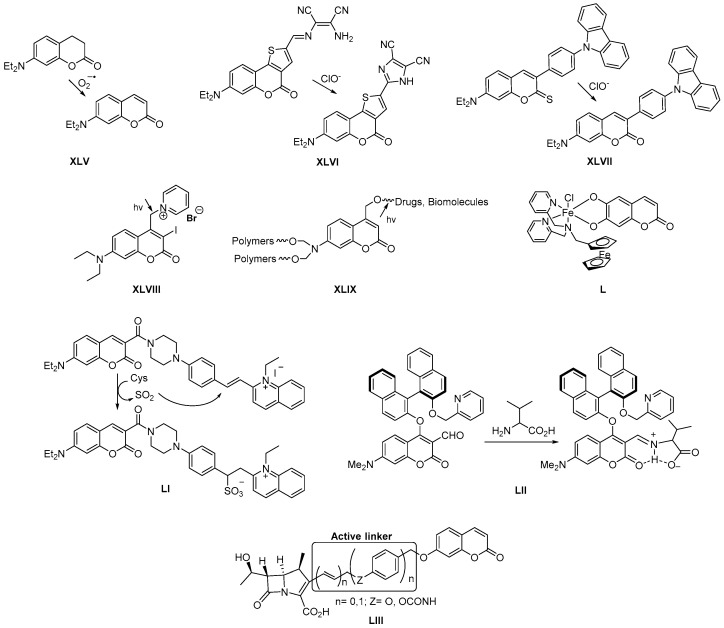
Structures of coumarin derivatives as fluorescent probes.

Coumarins are being used in the selective detection of metals such as copper (Figure 8, general structure **XXXVIII**) [60] or its determination in drinking water (Figure 8, compound **XXXIX**) [61,62]. A recent review focuses on the detection of iron in water and its applications [63]. Other works study the fluorescence determination of the presence of silver in aqueous medium (Figure 8, compound **XL**) [64]. In the case of mercury, there are also published works in which the selective determination in water is studied (Figure 8, general structure **XLI**) [65]. In some cases, this determination is selective, but in this case, the innovative methodology can be applied over a wide pH range (Figure 8, compound **XLII**) [66].

In some cases, these metal complexes serve as probes for the detection of biothiols, as in the case of copper complexes with benzothiazoles (Figure 8, compound **XLIII**) used in the determination of cysteines [67], or coumarin–quinoline complexes used in the detection of glutathione (Figure 8, compound **XLIV**) [68]. In other cases, aromatization to form the coumarin ring is used as a fluorescence test to detect the superoxide anion (Figure 8, compound **XLV**) [69].

In addition to the determination of metals, in many cases coumarin derivatives are used as fluorescence probes for the detection of hypochlorite, as in the case of coumarin–thiophene complexes (Figure 8, compound **XLVI**) [70] or of 2-thiocoumarins in which the presence of ClO– allows the formation of a fluorescent coumarin (Figure 8, compound **XLVII**) [71].

Coumarins can also be used as photocleavable linkers in the controlled release of drugs or biomaterials. This is the case of the in vivo photolysis of the microtubule inhibitor 4-pyridinomethylcoumarin (Figure 8, compound **XLVIII**) [72]. 7-Hydroxymethyl substituted aminocoumarins are used as iron complexes (Figure 8, general structures **XLIX** and **L**), and can be used to photochemotherapeutically target the mitochondria in the treatment of cancer [60,73].

Other reviews report on the use of 7-hydroxycoumarin and its derivatives in determining the activity of cytochromes P450 (CYP) enzymes [74] as well as 7-aminocoumarin derivatives in determining amino acids from serine or cysteine proteases [58]. In other cases, they are used to detect the metabolism of mitochondrial cysteines, the oxidation of which is a measure of cellular oxidative stress (Figure 8, compound **LI**) [75]. The use of the chiral coumarin-BINOL hybrid allows the enantioselective detection of amino acids (Figure 8, compound **LII**) [76]. Finally, coumarins can be used for easy detection of bacterial carbapenamases in which the coumarin fluorophore binds to the carbapenemic structure via a reactive linker (Figure 8, general structure **LIII**) [77,78].

## 3. Perspectives

Coumarins are privileged structures for biological applications. Their conjugated double ring system allows different spots for chemical modifications, and a large number of derivatives can be obtained. Therefore, structure/activity studies appear to be the hottest emerging topic. During the year 2020, more than a thousand research articles and reviews related to coumarins could be found. This highlights the great potential that these molecules can have in different fields of research. For simplicity, our overview focused on the potential of coumarins in medicinal chemistry. The most relevant studies were included. The range of applications described in this document, and some others outside the scope of this general description (i.e., optoelectronic applications [79], polymers [80], etc.), reflect the versatility of this scaffold.

In our opinion, the potential of coumarins as fluorescent probes appears to be the most promising field of research for the next few years, since several coumarin derivatives have shown great potential in prodrug degradation (drug release) and diagnostics.

Due to the length of this general overview, synthetic strategies for obtaining new coumarins have not been discussed in detail. To find information on the most recent synthetic pathways, we recommend the manuscripts by Molnar and co-authors [81] and Kovač and co-authors [82], both from 2020. To find an overview of the wide range of biological activities of coumarins, a 2020 review by Pinto and co-authors [1] is strongly recommended. Finally, to find information on the most recent analytical methods (fundamentals, instrumentation, purification and quantification applications, optimization of experimental conditions, emerging ecological methods, etc.) we recommend the review by Xue-song and co-authors [83].

## 4. Conclusions

Coumarins belong to a privileged family for biomedical proposes. Their simplicity, chemical properties, and the efficiency of the synthetic routes to obtain a wide range of substitution patterns make these compounds highly attractive and versatile for medicinal and biological chemists. To date, and during 2020, more than a thousand research articles and reviews containing information on coumarins appear in the PubMed, SciFinder, and Mendeley databases. The number of research groups working on this scaffold, and the impact of the results, highlight the potential of these molecules. Special attention has been paid to the potential of coumarins in drug design, as well as to fluorescent probes. This last application seems to be the most promising field of research for the next few years, since several coumarin derivatives have shown great potential in the decaging of prodrugs (drug release) and for diagnostic purposes.

## Data Availability

Data presented is original and not inappropriately selected, manipulated, enhanced, or fabricated.
